# ANTIBIOTIC RESISTANCE: Biofilm Dispersing Agent Rejuvenates Older Antibiotics

**Published:** 2010-07

**Authors:** Carol Potera

**Affiliations:** **Carol Potera**, based in Montana, has written for *EHP* since 1996. She also writes for *Microbe, Genetic Engineering News*, and the *American Journal of Nursing*

An estimated 75% of bacterial infections involve biofilms, surface-attached colonies of bacteria that are protected by an extracellular matrix.^1^ Bacteria protected within biofilms are up to 1,000 times more resistant to antibiotics than if they were free-floating (planktonic),^2^ which severely complicates treatment options. Rather than searching for better antibiotics, researchers have discovered that small molecules^3^ known as 2-amino-imidazoles disrupt biofilms, making antibiotic-resistant strains of bacteria more vulnerable to conventional drugs.^4^ Moreover, antibiotics enhance the ability of 2-amino-imidazoles to disrupt biofilms. “Perhaps new antibiotics are not the only way to combat biofilm infections if we could make ineffective older antibiotics active again,” says principal investigator Christian Melander, an associate professor of bio-organic chemistry at North Carolina State University.

Melander and his colleagues started with natural 2-amino-imidazoles (isolated from sponges) including oroidin and ageliferin, which are known to block biofilm formation. They synthesized an improved version of oroidin, 2-amino-imidazole/triazole (2-AIT), which featured more powerful antibiofilm activity and less toxicity to surrounding human cells and organs.^5^ Although 2-AIT alone does not kill bacteria, it disperses biofilms, releasing planktonic cells that are more susceptible to antibiotics than bacterial cells shielded by sticky biofilms.

The researchers tested 2-AIT against a variety of clinically relevant biofilm infections along with antibiotics that currently are used or have been used in the past to treat them. Multidrug-resistant strains of *Acinetobacter baumannii* plague soldiers wounded in the Middle East, and colistin, an older antibiotic with toxic side effects, remains a treatment of last resort due to extensive side effects.^6^ *Staphylococcus aureus* infections that colonize catheters and other indwelling medical devices were treated with the antibiotic novobiocin until drug-resistant *S. aureus* strains arose.^7^ Tobramycin is an inhaled antibiotic currently used to treat *Pseudomonas aeruginosa* infections that clog the lungs of cystic fibrosis patients.^8^ “We chose three antibiotics known to act against certain bacteria circulating in hospital settings that have become drug resistant,” says Melander.

The biofilms were grown in culture, then treated with their corresponding antibiotic, all of which produced little dispersion. However, the addition of 2-AIT to the antibiotics produced dramatic dispersion of up to 1,000-fold.^4^ Like adjuvants that boost the power of vaccines, 2-AIT is “our version of a small molecule adjuvant that allows several classes of older antibiotics to work again,” says Melander. He suspects 2-AIT somehow short-circuits bacterial signaling pathways that regulate biofilm formation; experiments are under way to unravel the details.

2-AIT also was shown to resensitize drug-resistant bacteria to death by antibiotic. When a clinical strain of methicillin-resistant *S. aureus* (MRSA) was treated with 2-AIT alone, it grew normally. The addition of methicillin, however, reduced its growth by 99%. Additionally, 2-AIT lowered the amount of antibiotics needed to inhibit bacterial growth.^4^

The combination of 2-AIT with antibiotics could serve as a parallel treatment for antibiotic-resistant infections. The results suggest this cooperative approach may enable “obsolete antibiotics to overcome infections that otherwise would persist if treated with either agent individually,” says Melander. A small molecule adjuvant like 2-AIT potentially could be given orally in pill form, he says.

2-AIT “most remarkably can actually disperse preformed biofilms, something that is much more difficult to do than simply inhibiting their formation,” says Neville Kallenbach, a professor of chemistry at New York University in New York City. Because biofilms are much harder to kill than planktonic bacteria, the combination therapy opens a new avenue for remediating persistent biofilm infections. “The ability to disperse biofilms formed by multidrug-resistant bacteria adds a major new weapon to the limited arsenal of therapies available today,” says Kallenbach—and the impact on human health could be enormous.

Agents such as 2-AIT also lend themselves to solving environmental biofilm problems including the biofouling of ship hulls and plugging of waterlines by microbes such as *Escherichia coli* and *Bacillus*, *Pseudomonas*, *Proteobacter*, and *Actinobacteria* species. Today’s antifouling paints contain copper, which leaches into seawater, where it inhibits enzymatic activity in brine shrimp larvae^9^ and impairs sperm quality and larvae development in sea urchins,^10^ among other effects. Melander is working on a copper-free 2-AIT-based polymer spray to prevent biofouling.

## Figures and Tables

**Figure:**
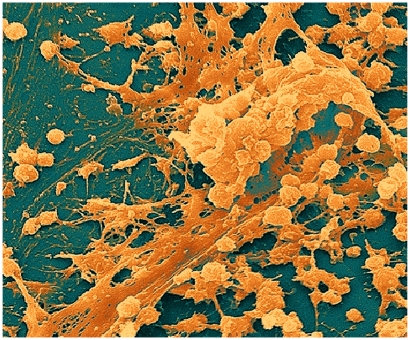
*Staphylococcus* biofilm on the inner surface of a needle
